# Effect of surface coating with seeds mucilages and xanthan gum on oil uptake and physical properties of fried potato strips

**DOI:** 10.1002/fsn3.2583

**Published:** 2021-09-13

**Authors:** Fakhreddin Salehi, Amirreza Roustaei, Alireza Haseli

**Affiliations:** ^1^ Faculty of Agriculture Bu‐Ali Sina University Hamedan Iran

**Keywords:** color changes, deep‐fat frying, modeling, seeds gum, surface shrinkage

## Abstract

The oil uptake and high‐fat/oil content problem associated with fried food products can be decreased using polysaccharides (mucilages and gums) as edible coatings. In this study, the efficiency of seeds mucilages (Balangu, Basil, and Wild sage), and xanthan gum as edible coatings on the oil uptake, moisture retention, and physical properties (color indexes and surface shrinkage) of potato strips during oil frying have been examined. Coating treatment with seeds mucilages and xanthan gum significantly decreased the moisture loss and oil uptake of potato strips (*p* < .05). Lightness (L*) index values of uncoated‐control and coated potato strips decreased during frying. The lowest and highest color change intensity (∆*E*) and surface shrinkage (%) values were observed for the potato strips coated by Wild sage seeds gum and uncoated‐control sample, respectively. The MMF (Morgan‐Mercer‐Flodin) model was selected as the best equation for describing the color changes intensity kinetics of untreated and treated potato strips during frying. The appropriate coating suspension for reducing the oil content of fried potato strips and improving product appearance quality was 1.0% Wild sage seeds gum.

## Graphical abstract

In this study, the efficiency of Balangu seeds, Basil seeds, Wild sage seeds, and xanthan gums as edible coatings on the oil uptake, moisture retention, and physical properties of fried potato strips was studied. The appropriate coating suspension for reducing the oil content of fried potato strips and improving product appearance quality was 1.0% Wild sage seeds gum.

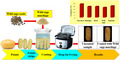

## 1 INTRODUCTION

Frying remarkably increases energy content of food products through oil uptake, and frequent consumption of fried products has been associated with health complications like obesity, cardiovascular diseases, and diabetes. So, there has been intense interest in decreasing the oil uptake during oil frying of foods. Gums‐based edible coatings offer as technique to formation a barrier to oil uptake during the frying process (Akdeniz et al., 2005; Albert & Mittal, 2002; Salehi, 2020).

Various edible coating materials have been utilized in diverse fried food products to improve some quality attributes (Salehi, 2020). Balangu (*Lallemantia royleana* L.), Basil (*Ocimum basilicum* L.), and Wild sage (*Salvia macrosiphon* L.) are the pharmaceutical native plant which attracted a high level of interest to be used as an edible coating is grown mostly in various regions of Asia, Europe, and Middle East, chiefly in different regions of Iran. These plants seeds contain considerable amounts of mucilage (gum), while soaked in water, could provide some of favored technological functions for several fried food products. Some functional properties of the extracted gums that are commonly known as Balangu, Basil, and Wild sage seed gums including biodegradable properties, eco‐friendliness, low extraction and production cost, accessibility, hydrophilic nature, and appropriate rheological characteristics caused high interest to more application as coating material (Amini et al., 2021). These gums have the suitable physical properties as an edible coating for enhancement of appearance quality of fried food products. Also, these seed gums are able to lower the migration of lipids (Salehi, 2020).

The surface color and appearance quality of the fried food products are one of the highest significant quality factors for the acceptance of these products. Also, the surface color measurements of fried foods (during frying procedure) can be used in an indirect way to determine the product appearance change, since they are simpler and faster than chemical analysis (Salehi, 2018, 2020). Kurek et al. (2021) investigated on the efficiency of coatings treatment with carboxymethyl cellulose, chitosan, pectin and arabic gum, and natural antioxidants on fresh‐cut potato's color, pH, moisture content, stability, and oil uptake after deep fat frying. The authors reported that the coatings significantly decreased oil content in deep fat fried potato strips, without effects on L*, b*, whiteness index, and Δ*E*. In addition, Kizito et al. (2017) reported that the hydrocolloid coatings (carboxymethyl cellulose, pectin, agar, and chitosan) of potato strips decreased the quantity of oil uptake by samples by 12.93% (pectin), 11.71% (carboxymethyl cellulose), 8.28% (chitosan), and 5.25% (agar). Also, hydrocolloid coating treatments and concentration (1 and 2%) significantly decreased moisture loss of strips (*p* < .05).

Process variables include fryer type, frying temperature and condition, sample pretreatment, and edible coatings are expected to affect the color and surface of the fried products (Salehi, 2020). While there are many literature studies on the oil uptake and moisture content of uncoated fried food products, no studies related to the moisture content, oil uptake, color indexes change (L*, a*, b*, and Δ*E*), and surface area change during frying of coated potato strips with seeds gums (Balangu, Basil, and Wild sage) were found in the literature. Therefore, the objective of this work is to examine the moisture content, oil uptake, and kinetics of color change and shrinkage during frying of coated potato strips with Balangu seeds, Basil seeds, Wild sage seeds, and xanthan gums.

## 2 MATERIALS AND METHODS

### 2.1 Potato strips preparation

Potatoes were harvested in a patch located in Hamedan, Iran. Potatoes were hand‐peeled, cut with a oval‐shaped mold (strips), and knife into strips with 1 cm thickness.

### 2.2 Seeds gum powder preparation

The cleaned Balangu seeds, Basil seeds, and Wild sage seeds were firstly immersed in distilled water at the seed/water ratio of 1:20, at 25℃, and for 20 min. Gums extract was separated from the swollen seeds by passing the seeds through a extractor equipped (Panasonic, MJ‐J176P). The extracted gums were then dried in an air‐forced oven (Shimaz) at 60℃, and finally, the gums powder was milled, sieved, packed, and kept in dry condition at 20℃ (Amini et al., 2021; Satorabi et al., 2021).

### 2.3 Coating of potato strips

**FIGURE 1:**
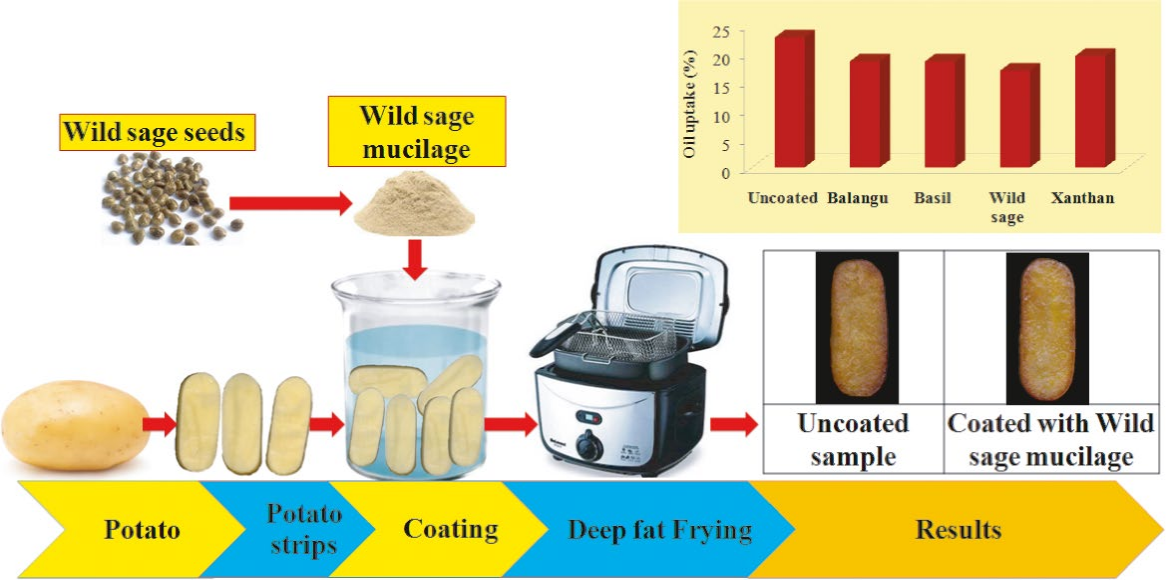
Schematic of coating and frying process of potato strips

Edible coating is the one of the ways to decreasing the oil uptake of fried potato strips. In this study, Balangu seeds, Basil seeds, Wild sage seeds, and xanthan gums were used to coating the fresh potato strips before frying. A 1.0% (w/w) of each gums solutions were prepared at 25℃, and then potato strips were immersed for 1 min in these solutions (Figure 1).

### 2.4 Frying condition

Fresh refined sunflower oil (Bahar frying oil) was used as a frying medium for each frying batch. After potato strips were soaked in gums solutions for 60 s, they were inserted to the sample holder, which were then immersed in hot oils in a mini fryer (Delmonti, DL630). They were followed by frying at 180ºC for 1–12 min and cooling down at 20℃ for 4 min. Also, K‐type thermocouples (thermocontroller, ±0.1℃, Lutron, TM‐916) with a 1‐s response time were used in measuring temperatures.

The weight changes of the potato strip samples were measured using a digital balance (LutronGM‐300p) with an accuracy of ±0.01 g. The values of total solid and moisture content (MC) of fresh and fried potato strips were measured using an oven (Shimaz) at 105℃ for 5 hr (using Equation 1). The average moisture content of fresh potato strips was 79%.

(1) $$\text{MC}=\frac{M_{1}‐M_{2}}{M_{1}}\times 100$$

In this equation, MC is the moisture content (%), *M*_1_ is the initial weight of the samples (before oven) (g), and *M*_2_ is the final weight of samples (after oven) (g). Also, the oil uptake (%) of fried potato strips was calculated using Equation 2:

(2) $$\text{OU}=\frac{{\text{TS}}_{2}‐{\text{TS}}_{1}}{M_{1}}\times 100$$

where OU is the oil uptake (%), TS_2_ is the total solid of fried potato strips (g) (the final weight of fried potato strips, after oven), TS_1_ is the total solid of fresh potato strips (g), and *M*_1_ is the weight of each fried potato strip (g).

### 2.5 Color and shrinkage

The samples’ photos were captured with a scanner (HP, Scanjet‐300). Color and shrinkage (area) of fresh and fried potato strips (uncoated‐control and coated samples) were analyzed using ImageJ software (version 1.42e). Color scale, namely L* (lightness), a* (redness), and b* (yellowness), of these samples was determined (Salehi, 2017). The Δ*E* (color changes intensity) values for the total color difference were determined using Equation 3:

(3) $$\Delta E=\sqrt{{\left(\Delta L^{*}\right)}^{2}+{\left(\Delta a^{*}\right)}^{2}+{\left(\Delta b^{*}\right)}^{2}}$$

The shrinkage (area) change of the potato strips during frying procedure was determined using Equation 4:

(4) $$\Delta A=\frac{A_{0}‐A_{t}}{A_{0}}\times 100$$

where Δ*A* is the shrinkages change (%), and *A*_0_ and *A_t_* (cm^2^) are the area of the fresh potato strips and the fries samples at frying period of *t*, respectively.

### 2.6 Mathematical modeling

In this study, the power, quadratic, and sigmoidal models (Gompertz relation, logistic model, Richards model, MMF (Morgan‐Mercer‐Flodin) model, and Weibull model) were selected to characterize the color changes intensity within the frying process of uncoated‐control and coated potato strips with Balangu seeds, Basil seeds, Wild sage seeds, and xanthan gums (Hyams, 1997; Salehi, 2019; Salehi & Satorabi, 2021).

### 2.7 Statistical analysis

Significant difference between data means was estimated using Duncan's multiple range test at *p*‐value<0.05 (SAS 9.1 Institute, Inc.), and it was carried out to establish the impact of edible coatings (coating with Balangu, Basil, and Wild sage seeds mucilages and xanthan gum) on the moisture content, oil uptake, color indexes (a*, b*, L*, and ∆*E*), and surface area changes of fried potato strips (*p*‐value < .05). The results will be compared with uncoated‐control potato strips. All frying experiments and samples analysis were carried out in triplicate.

## 3 RESULTS AND DISCUSSION

**FIGURE 2:**
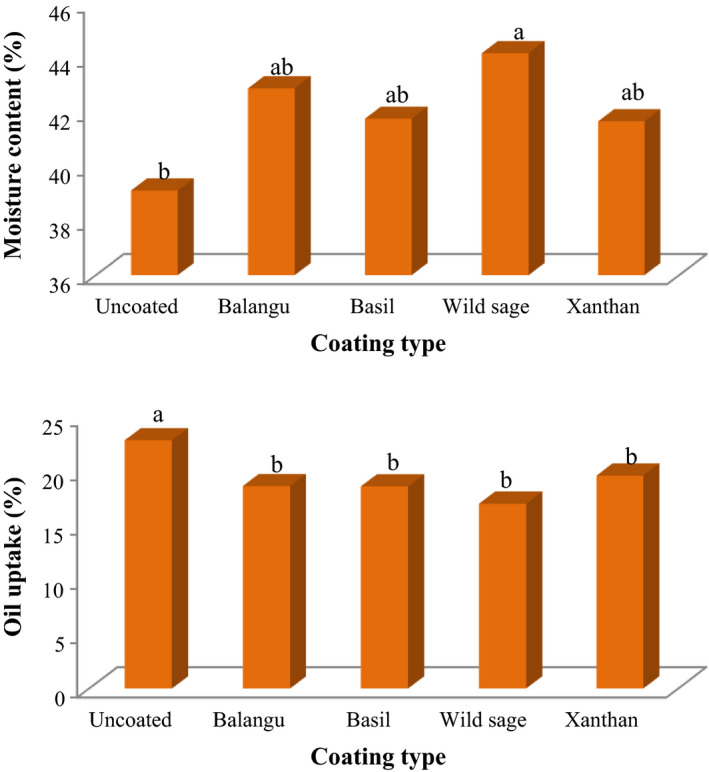
Influence of edible coatings on the moisture content and oil uptake of fried potato strips

It is generally recognized that oils are absorbed in food products during frying by water evaporation as well as water vapor condensation. Edible coatings applied to foods before frying are a technique that can increase the quality and overall acceptability of fried potato strips. Figure 2 demonstrates the influence of coatings with Balangu seeds, Basil seeds, Wild sage seeds, and xanthan gums on the moisture content and oil uptake of fried potato strips. The moisture content was 39.12 for the uncoated‐control samples, while 42.87, 41.75, 44.17, and 41.66 were determined for fried samples coated with Balangu seeds, Basil seeds, Wild sage seeds, and xanthan gums, respectively. The coated potato strips had high moisture content due to the inhibitory influences of the seeds gums and xanthan gum compared to the untreated samples. The results showed that seeds gums and xanthan gum could increase moisture retention and decrease oil uptake of deep fried potato strips (*p* < .05). There was no significant difference between coated potato strips with seeds gums and xanthan gum in oil uptake (*p* > .05). Naji Tab asi and Mahdian (2017) and Karimi and Kenari (2016) also observed a similar trend of low oil uptake during frying of treated potato chips with Persian gum and treated potato strips with Basil seed gum and salep, respectively. Khazaei et al. (2016) observed that the treatment with Basil seed gum and thymol improved the appearance of the fried shrimps and also the least oil uptake and the highest moisture content in the total frying duration and at different condition were reported for these coated samples. Also, Eslampour and Hosseini (2017) observed that the coating with bitter almond gum and gelatin increased moisture content and decreased oil uptake of fried potato slices. Bouaziz et al. (2016) observed that the coating with almond gum improved the appearance and quality of the fried potato chips and also the least oil uptake and the highest moisture content in the total frying duration and at different condition were reported for these coated samples. The influence of various treatments and frying times on physicochemical and sensorial properties of potato balls coated with chickpea and wheat flour was studied by Kilincceker and Hepsag (2012). The authors reported that the breading materials raised the yield values compared to the control, the yield, and moisture content values reduced with increasing frying time. In another study, application of transglutaminase‐crosslinked whey protein/pectin films as water barrier coatings in fried doughnuts and French fries and also baked food like “taralli” biscuits were studied by Rossi Marquez et al. (2014). Their results showed a significant reduction in oil content in the coated fried foods (about 50% in doughnuts and 25% in French fries) with respect to both uncoated‐controls and whey/soy protein‐coated samples.

**FIGURE 3:**
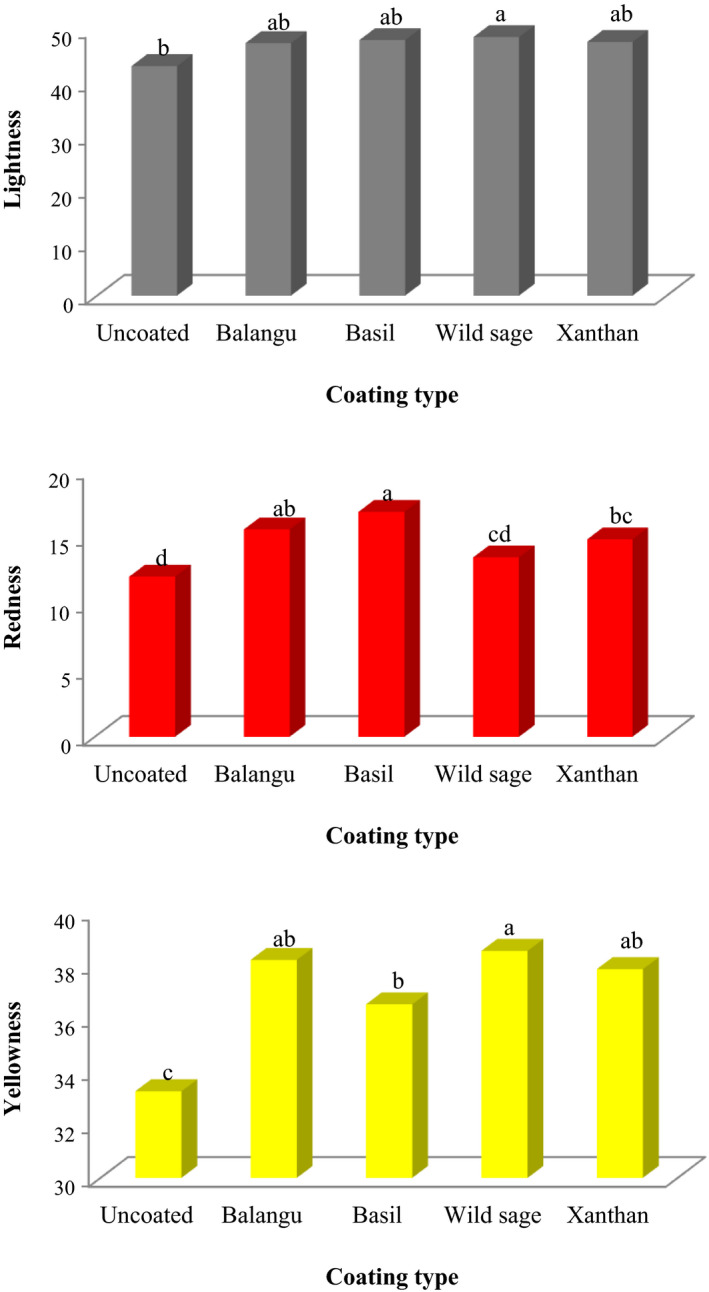
Influence of coatings on the color parameters (lightness, redness, and yellowness) of fried potato strips

The final color of the fried foods depends on the pretreatment of fresh samples, surface coating, frying condition, fryer temperature, frying duration, and also the oil uptake of samples. Figure 3 demonstrates the influence of edible coatings on the L*, a*, and b* values of fried potato strips. The color indexes of the fried potato strips containing Balangu seeds, Basil seeds, Wild sage seeds, and xanthan gums treatments were better than the potato strips without any treatment. L* (brightness) is a main factor in the fried foods as it is universal the first quality attribute evaluated by user when assessing the products' acceptability. The loss of L* values gives the darker color and appearance of the fried products (Seerangurayar et al., 2019). The L* index values of fried potato strips reduced during frying, while rates were low for the same condition in the treated samples. The results showed that treatments have significant effects on the L* parameter (*p* < .05). The highest L* value was 48.51 for the coated potato strips with Wild sage seeds gum. Bouaziz et al. (2016) used almond gum as an edible coating to enhance the quality characteristics of potato chips during frying process. The authors reported that the coated slices have a significantly lighter color (high L* index) and were significantly yellower (high b* index) than the uncoated products. The L* value was 58.2 for the uncoated slices, while it was 88.3 and 87.5 for fried slices coated with almond gum and arabic gum, respectively.

The a* value demonstrates redness for fried products, and the effect of the types of treatment on the a* values of fried potato strips is demonstrated in Figure 3. We observed that the a* index value was an indicator of browning (color changes) during frying of potato strips. Also, a* index values of uncoated‐control and coated potato strips increased during frying, while rates were low for the same condition in the uncoated‐control sample (*p* < .05). The a* value was 12.03 for the uncoated‐control sample, while 15.56, 16.88, 13.46, and 14.82 were found for fried samples treated with Balangu seeds, Basil seeds, Wild sage seeds, and xanthan gums, respectively.

Yellowness (b*) is the most desired color attribute in fried products. The results showed that coatings with seeds gums and xanthan gum have significant effects on the b* parameter (*p* < .05). The highest and lowest b* values were observed for the potato strips coated by Wild sage seeds gum and uncoated‐control sample, respectively. The b* value was 33.25 for the uncoated‐control sample, while 38.19, 36.52, 38.53, and 37.84 were found for fried samples treated with Balangu seeds, Basil seeds, Wild sage seeds, and xanthan gums, respectively. Kizito et al. (2017) studied on the properties of potato strips as affected by different hydrocolloid coatings (carboxymethyl cellulose, pectin, agar, and chitosan), frozen storage, and frying conditions. The authors reported that the L* values of coated samples were equivalent to the untreated samples (58.99) except chitosan‐coated strips (51.75). Also, a* values of treated samples were not significantly different from the untreated samples (9.59) except agar‐treated strips (6.51).

**FIGURE 4:**
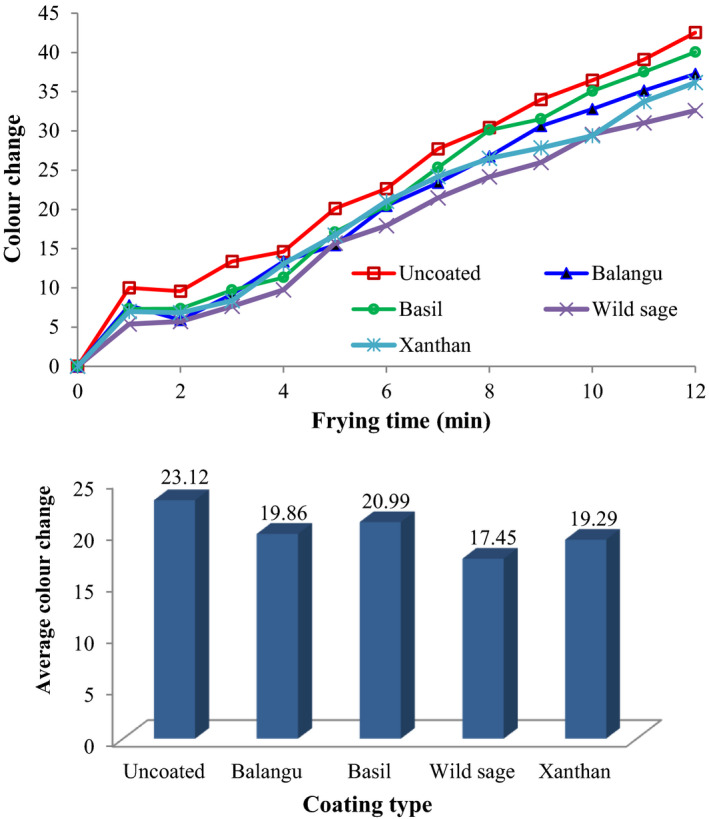
Influence of coatings type and frying time on the color change intensity of fried potato strips

In Figure 4, color change intensity (Δ*E*) values were reported as function of frying duration and edible coatings type. As shown in Figure 4, the Δ*E* values of uncoated‐control and coated potato strips increased during frying process, while rates were low for the same condition in the treated strips. In addition, Figure 4 demonstrates the influence of the types of treatment on the average color change intensity (∆*E*) of fried potato strips. The lowest and highest ∆*E* values were observed for the potato strips coated by Wild sage seeds gum and uncoated‐control sample, respectively. The average color change intensity value was 23.12 for the untreated strips, while 19.86, 20.99, 17.45, and 19.29 were found for fried strips treated with Balangu seeds, Basil seeds, Wild sage seeds, and xanthan gums, respectively.

Kinetics modeling of color change intensity is necessary tools for the optimization of frying conditions and controlling or improving the process to get high quality of the fried products. The various equations were fitted to the color change intensity values and the calculation of the parameters, resulted from fitting models to the empirical data. The results of fitting the MMF model (Equation 5) to the empirical data are collected in Table 1.

(5) $$\Delta E=\frac{ab+ct^{d}}{b+t^{d}}$$

where Δ*E* and t are the color changes intensity and frying duration, respectively, and *a*, *b*, *c*, and d are the coefficients of the models. The highest correlation coefficient (*r*) and lowest standard error (SE) values of the estimate of fitting showed that the color change during frying of uncoated‐control and coated potato strips could be modeled by the MMF model.

**TABLE 1:** The MMF model coefficients for color change index of potato strips during frying

Coating type	*a*	*b*	*c*	*d*	SE	*r*
Uncoated‐control	0.0653	18.7275	91.1570	0.6182	0.467	0.997
Balangu seeds	−0.0083	9.7485	35.4264	0.9064	0.581	0.995
Basil seeds	0.1596	13.2991	47.2384	0.7933	0.574	0.995
Wild sage seeds	0.0337	159.0094	540.8916	0.5875	0.356	0.997
Xanthan	0.1254	7.1171	24.8209	0.9673	0.444	0.997

**FIGURE 5:**
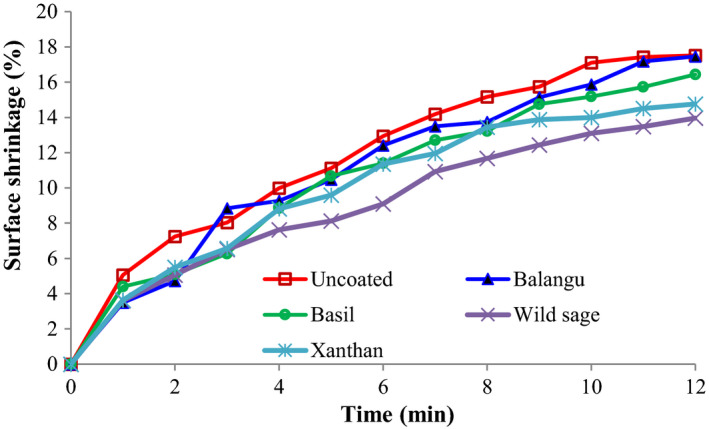
Influence of coatings on the surface shrinkage of potato strips during frying process

In Figure 5, shrinkage of uncoated‐control and coated potato strips was demonstrated as functions of frying duration and the types of treatment. The surface shrinkage (%) of uncoated‐control and coated potato strips was increased with the progression of frying duration, while rates were low for the same condition in the coated potato strips with Wild sage seeds gum. The average surface shrinkage value during frying process of potato strips was 11.66% for the untreated strips; in comparison, 10.93%, 10.36%, 8.89%, and 9.84% were found for fried potato strips coated with Balangu seeds, Basil seeds, Wild sage seeds, and xanthan gums, respectively. The lowest average surface shrinkage was seen in coated potato strips with Wild sage seeds gum (8.89%).

## 4 CONCLUSION

The influence of Balangu seeds, Basil seeds, Wild sage seeds, and xanthan gums as edible coatings on the oil uptake and color change kinetics of potato strips during frying has been studied. The L* index values decreased, but the a* index values increased during frying. There was a significant difference in L* values (surface brightness) when comparing the uncoated and coated potato strips. Coated potato strips with Wild sage seeds gum presented the highest visual brightness (the highest L*) compared to the other coated and uncoated strips. In addition, these strips were yellower (higher b*) than the untreated strips (*p* < .05). The lowest color change intensity value was used for the coated potato strips with Wild sage seeds gum. Various kinetic equations were used to fitting the experimental data, and the results showed that the MMF model was the best at describing the color change intensity curve with the average correlation coefficient equal to 0.996 and the average standard error equal to 0.484. The surface shrinkage (%) of uncoated‐control and coated potato strips was increased during frying time, while rates were low for the same condition in the coated potato strips with Wild sage seeds gum. The appropriate coating for decreasing the oil content of fried potato strips and improving product appearance quality was 1.0% Wild sage seeds gum.

## CONFLICT OF INTEREST

We have no conflict of interest to declare.

## ETHICAL APPROVAL

This study does not involve any human or animal testing.

## INFORMED CONSENT

Written informed consent was obtained from all study participants. The manuscript is not submitted or under consideration in any other journals.

## Data Availability

All data generated or analyzed during this study are included in this published article.
